# Level of Orthographic Knowledge Helps to Reveal Automatic Predictions in Visual Word Processing

**DOI:** 10.3389/fnins.2021.809574

**Published:** 2022-02-09

**Authors:** Zehao Huang, Shimeng Yang, Licheng Xue, Hang Yang, Yating Lv, Jing Zhao

**Affiliations:** ^1^Jing Hengyi School of Education, Hangzhou Normal University, Hangzhou, China; ^2^Zhejiang Key Laboratory for Research in Assessment of Cognitive Impairments, Hangzhou, China; ^3^Center for Cognition and Brain Disorders, Hangzhou Normal University, Hangzhou, China; ^4^Institute for Brain Research and Rehabilitation, South China Normal University, Guangzhou, China; ^5^Key Laboratory of Brain, Cognition and Education Sciences (South China Normal University), Ministry of Education, Guangzhou, China; ^6^Faculty of Science and Engineering, Bernoulli Institute for Mathematics, Computer Science and Artificial Intelligence, University of Groningen, Groningen, Netherlands

**Keywords:** orthographic knowledge, N170, EEG, color matching task, visual word processing, predictive coding

## Abstract

The brain generates predictions about visual word forms to support efficient reading. The “interactive account” suggests that the predictions in visual word processing can be strategic or automatic (non-strategic). Strategic predictions are frequently demonstrated in studies that manipulated task demands, however, few studies have investigated automatic predictions. Orthographic knowledge varies greatly among individuals and it offers a unique opportunity in revealing automatic predictions. The present study grouped the participants by level of orthographic knowledge and recorded EEGs in a non-linguistic color matching task. The visual word-selective N170 response was much stronger to pseudo than to real characters in participants with low orthographic knowledge, but not in those with high orthographic knowledge. Previous work on predictive coding has demonstrated that N170 is a good index for prediction errors, i.e., the mismatches between predictions and visual inputs. The present findings provide unambiguous evidence that automatic predictions modulate the early stage of visual word processing.

## Introduction

Efficient processing of written words is crucial to reading, a cognitive ability humans need in daily living. For written word processing, the brain not only receives bottom-up visual inputs but also generates top-down predictions that help to resolve the conflicts between visual inputs and internal lexical knowledge (Price and Devlin, 2011). Following the predictive coding theory (Hinton, 2007; Friston, 2010), Price and Devlin (2011) suggested the ventral occipitotemporal area (vOT) as a bridge that connects bottom-up visual inputs, i.e., written words, and top-down predictions, i.e., priori associations between visual inputs and phonology and semantics (McCandliss et al., 2003; Devlin et al., 2006; Reinke et al., 2008; Woodhead et al., 2011). More specifically, words and word-like stimuli would quickly activate the associated phonology and semantics, which are fed to vOT to facilitate the processing of visual inputs. Known as the “interactive account,” this theory has gained support from neuroimaging studies, most notably, the study of the left-lateralized N170 event-related potential (ERP) component (or the N1 component in some studies). N170 is the first negative ERP component recorded at posterior electrodes. It arises from the occipitotemporal area and it has been used to index neural activation in vOT (Rossion et al., 2003; Brem et al., 2009). Widely recognized as an electrophysiological marker of orthographic processing, the N170 component is stronger in response to words than to non-words (e.g., Maurer et al., 2005; Zhao et al., 2012; Eberhard-Moscicka et al., 2015).

One important aspect of the “interactive account” is that it makes a distinction between top-down (strategic) and automatic (non-strategic) predictions. Strategic predictions are easily manipulated with task demands (e.g., Maurer et al., 2006; Lupker and Pexman, 2010). For instance, Wang and Maurer (2017) examined the effect of task-related strategic predictions in three tasks. The results revealed stronger N170 responses to symbols than to words in their delayed naming and color detection tasks, but not in the repetition detection task. Yum et al. (2014) examined phonological regularity in a lexical decision task and a delayed naming task. They found that phonologically regular Chinese characters elicited stronger N170 responses than irregular Chinese characters, but only in the delayed naming task, suggesting that top-down phonological predictions depend on task demands. While these studies provided clear evidence that top-down strategic predictions modulate visual word processing, empirical evidence for automatic predictions in visual word processing is still lacking.

Orthographic knowledge connects meaning and pronunciation (Liu et al., 2007), and it represents the legal position, the combination rules, etc., of character components in Chinese (Loh et al., 2018). It is typically measured with lexical decision tasks (e.g., Apel, 2011) where the researcher presents real or pseudo words (or visual symbols) and the participant indicates whether an item is a real word or not (e.g., Tong et al., 2009). Orthographic knowledge varies greatly among readers. While some people excel on the lexical decision task, others have great difficulties in correctly identify non-words (e.g., pseudo words; Davis et al., 2009; Yang et al., 2012; Taha and Azaizah-Seh, 2017). According to the interactive account (Price and Devlin, 2011), words and word-like stimuli would evoke predictions. Consequently, individuals who misclassify pseudo words would show stronger neural responses to pseudo than to real words. The reason being that pseudo words are not in the lexicon and as a result, the evoked predictions will not match the visual input, leading to prediction errors and stronger neural activation in vOT. In the same vein, those who correctly classify pseudo words as non-words would produce similar neural responses to pseudo and real words, because the pseudo words evoke no or few prediction errors. In line with these predictions, a recent study revealed stronger N170 responses to stimuli of low orthographic regularity (e.g., non-words), but only in young children with low orthographic knowledge (Zhao et al., 2019). The color-matching task used in Zhao et al. (2019) did not require any linguistic processing, so orthographic regularity was processed implicitly or automatically by the brain. To the best of our knowledge, this was the first neuroimaging study that unambiguously examined automatic predictions in visual word processing. However, this study only examined young children whose orthographic knowledge was still fast developing. For a proper examination of automatic prediction and its neural correlates in visual word processing, we need to test skilled adult readers whose lexicons are relatively stable.

The present study examined automatic predictions in visual word processing with a group of skilled Chinese adult readers. The readers were grouped based on their responses to pseudo Chinese characters, with one group more likely to misclassify pseudo words as real words. As discussed, pseudo words would evoke more prediction errors in those who are more likely to misclassify pseudo words as real ones, leading to stronger neural activations in vOT. Because explicit language tasks and long stimulus exposure time may recruit task-related predictions, a one-back color matching task with short stimulus exposure time was adopted. This color matching task has been widely used to study implicit word processing (Lin et al., 2011; Shtyrov et al., 2013; Xue et al., 2019) and the word-selective N170 response is frequently observed. In this task, the participants report the color of one stimulus rather than its content, so the processing of orthographic, phonological, and semantic information is implicit by nature (Lin et al., 2011; Zhao et al., 2012).

Based on the “interactive account” (Price and Devlin, 2011), we predict that pseudo words would evoke few prediction errors in participants who correctly classify pseudo words as non-word (high orthographic knowledge) and the N170 response should be similar to real and pseudo words. By contrast, pseudo words would evoke more prediction errors in participants who misclassify pseudo words as real ones (low orthographic knowledge), leading to stronger N170 responses to pseudo than to real words.

## Materials and Methods

### Participants

Fifty-two college students participated in this study. They were right-handed native Chinese speakers, who reported normal or corrected-to-normal visual acuity. Five participants were excluded from the analyses due to excessive noise in the EEG recordings. As a result, 47 participants (19 males, mean age = 20.6 years, SD = 2.2, range = 18–25 years) were included in the analyses.

In the present study, all participant completed a lexical decision task (see Zhao et al., 2019, for a detailed discussion), and the rate of misclassifying the pseudo characters was used to median-split the participants into two groups. For convenience, we will refer to those two groups as high and low orthographic knowledge groups, respectively. Participants in the high orthographic knowledge group were less likely to report the pseudo characters as real ones. There are 23 and 24 participants in high and low orthographic knowledge group, respectively.

### Materials

**FIGURE 1:**
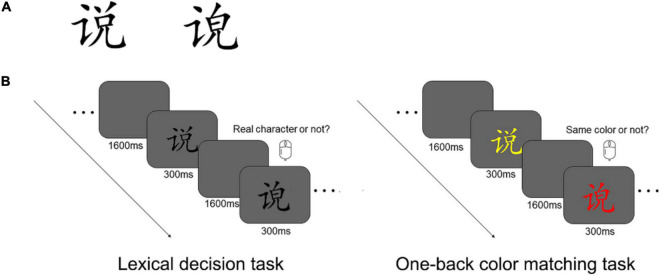
**(A)** Sample real (left) and pseudo (right) characters. **(B)** The lexical decision task (left panel) and the one-back color matching task (right panel). The lexical decision task was used to group the participants, whereas the color matching task was used to examine the neural responses associated with automatic predictions.

According to the Modern Chinese Frequency Dictionary (1985), the word frequency is usually in the 300–600 per million range. Similar to the research protocols of a previous study (Zhao et al., 2019), the present study only used Chinese characters with a “left-right” layout. In the Modern Chinese Dictionary, more than 80% of Chinese characters have a “left-right” layout. The other 20% of the characters are in other types of layouts (e.g., “up-down” and “semi-enclosing”). The stimuli used in the present study included both real and pseudo characters (see Figure 1A, for samples). Pseudo characters were created with the radicals from the same set of real characters, but they obviously do not exist in the dictionary. The real and pseudo characters were matched in terms of stroke numbers (ranging between 8 and 13) and structural complexity. The final set of stimuli consisted of 36 real and 72 pseudo characters. We had more pseudo characters in the tests because a pilot study showed that 36 pseudo characters were not enough to derive a reliable percentage measure of misclassification.

### Task Procedures

#### Lexical Decision Task

The lexical decision task was scripted with Python 3.6 and Pygame. All stimuli were presented on a 19-inch LCD monitor (HP l1908w), which had a maximum resolution of 1024 × 768 pixels and a refresh rate of 60 Hz. For better timing precision, stimulus presentation and response registration were controlled by a Windows PC equipped with a NVIDIA GT610 graphics card. Of the 90 items used in the lexical decision task, 30 items were real characters, and 60 items were pseudo characters. Stimuli were presented to the participant in a random order. The characters extended 1.5° × 1.5°, and they were presented in black against a gray background at the screen center. Each character was presented for 300 ms, followed by an ISI of 1450, 1525, 1600, 1675, or 1750 ms (Figure 1A). The participants reported whether the presented stimulus was a real Chinese character or not, by pressing the left and right button on a mouse. Both speed and accuracy were emphasized in this task. The response buttons were counter-balanced across the participants.

#### One-Back Color Matching Task

The one-back color matching task was programed with E-Prime 2.0, but the equipment used for stimulus presentation was identical to that used in the lexical decision task. Pseudo and real characters were inter-mixed and presented in a random order. Each character was presented in green, red, or yellow, against a gray background, at the screen center. The timing of trial events in the one-back color matching task matched that in the lexical decision task. The participant was instructed to press a key whenever a stimulus was in the same color as the previous one (i.e., one-back color repetition detection; see Figure 1B, right panel). To get enough trials for ERP averaging, each character was presented as the non-responding target for three times (for similar experimental manipulations, see Maurer et al., 2008; Cao and Zhang, 2011; Lin et al., 2011; Zhao et al., 2012, 2019). A subset of 12 pseudo and 6 real characters were presented three times as the responding target (on 16.67% of the trials). In total, the real characters were presented on 108 trials (90 as fillers and 18 as responding targets) and the pseudo characters were presented on 216 trials (180 as fillers and 36 as responding targets). The participants responded with either the left or right index finger (counter-balanced across participants).

The participants completed both tasks in a single session, and the lexical decision task was always carried out following the one-back color matching task.

### Electrophysiological Recording and Analysis

#### Data Acquisition and Pre-processing

EEG data were collected in the one-back color-matching task only. The EEG signal was recorded from 30 Ag/AgCl electrodes secured in an elastic cap according to the extended 10–20 system. The EEG data were recorded with a BrainAmp amplifier system and the software for EEG recording was Brain Vision Recorder (Brain Products GmbH, Germany). The Cz electrode served as an online reference, but the data were offline re-referenced to the average. Horizontal EOG was recorded in a bipolar lead from two additional electrodes placed on the outer canthi of the two eyes. Vertical EOG was recorded in a bipolar lead from additional electrodes placed on the supra-orbital and infra-orbital ridges of the right eye. The electrode impedance was kept below 5 kΩ. The EEG and EOG signal were continuously recorded and amplified at a sampling rate of 1000 Hz, with a band pass from AC 0.1 to 100 Hz.

In the offline analysis, the EEG signal was first low-pass filtered at 30 Hz and automatically scanned for artifacts in EEGLab (Delorme and Makeig, 2004), an open-source EEG data analysis toolbox implemented in MATLAB. Using the default parameters of the SASICA toolbox (Chaumon et al., 2015), eye movement artifacts in the EEG signal were removed with an ICA-based procedure (Delorme et al., 2012).

#### Event-Related Potential Analysis

**FIGURE 2:**
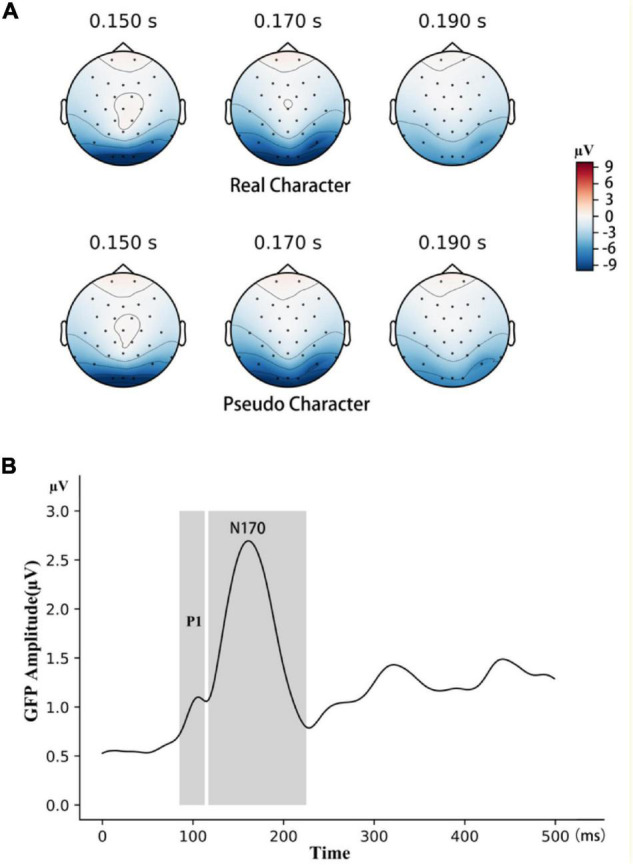
**(A)** The topographic maps evoked by real and pseudo characters, 150, 170, and 190 ms following stimulus presentation. **(B)** The time windows for P1 and N170 was selected with the GFP method, see text for details.

In the ERP analysis, continuous EEG data were epoched and baseline-corrected with a 200-ms pre-stimulus period. The post-stimulus period was 500 ms. For both pseudo and real characters, a trial was included in the analysis only if the character was not a target and no false positive response was issued. Before averaging, segments with artifacts exceeding ±80 μV (about 3% of all trials) were automatically rejected. For ERP averaging, on average, there were 86 real-character trials and 173 pseudo-character trials. Electrodes P7 and P8 were selected to analyze the N170 component, based on the topographic maxima of the negative field in the occipitotemporal area (see Figure 2A). These two electrode sites were frequently used to measure the N170 component in previous studies as well (e.g., Bentin et al., 1999; Maurer et al., 2008; Lin et al., 2011). The time windows for the P1 (51–135 ms) and the N170 (137–267 ms) components were selected using the GFP method (see Figure 2B); the selected time windows were similar to those reported in previous studies (Maurer et al., 2005; Cao and Zhang, 2011; Zhao et al., 2019). The peak amplitude of the P1 and N170 components were the mean voltages within a 20-ms window centered around the local maximum. For statistical analysis, Cohen’s d and partial eta squared (η*_*p*_*^2^) were reported as the effect size measures for *t*-tests and ANOVAs, respectively.

## Results

### Behavioral Data

#### The Lexical Decision Task

**FIGURE 3:**
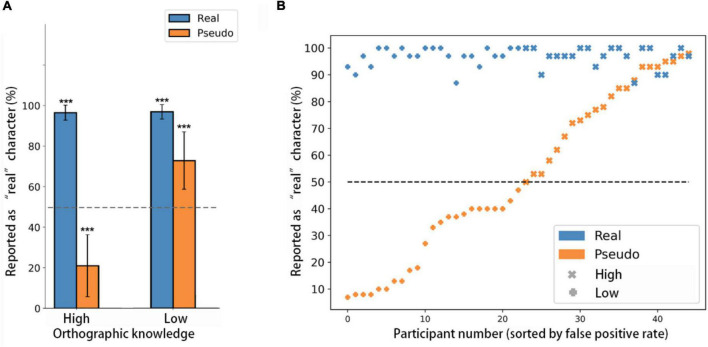
**(A)** The rate in reporting a stimulus a as real character in the high and low orthographic knowledge groups in the lexical decision task. **(B)** Same as **(A)** but showing data points from individual participant. ***p < 0.001.

The rate in reporting pseudo characters as real ones (false positives) was used to median-split the participants into high and low orthographic knowledge groups (see Figure 3A). A *t*-test on the false positive rates revealed a significant group difference, *t*(1,45) = 11.59, *p* < 0.001, Cohen’s *d* = 3.47. We also examined the rate in correctly reporting real characters (hits) but found no between-group difference, *t*(1,45) = 0.51, *p* = 0.61, Cohen’s *d* = 0.37.

To examine if there was a systematic response bias, we tested the rate in reporting a stimulus as a real character against chancel level with a binomial test (Bonferroni corrected). As is clear from Figure 3A, the rate in correctly identifying real characters was well above chance level in both groups (all *p* < 0.001); however, the rate in reporting pseudo characters as real characters was below chance level in the high orthographic knowledge group (*p* < 0.0001) and above chancel level in the low orthographic group (*p* < 0.001). As shown in Figure 3B, the data points from all participants also corroborate with these results, showing that the rate in reporting pseudo characters as real ones varied greatly among the participants, whereas the rate in correctly reporting real words did not vary much among the participants.

#### The One-Back Color Matching Task

For the color-matching task, we first examined the target hit rates and the response times (RTs). As is clear from Table 1, the hit rate was well above 90%. Analysis of the RTs, with variables Stimulus (real vs. pseudo characters) and Group (high vs. low orthographic knowledge), revealed a significant main effect of Group, *F*(1,45) = 4.85, *p* = 0.04, η_*p*_^2^ = 0.09, but no other main effect or interaction was significant, all *F*’s < 1, all *p*’s > 0.57, all η*_*p*_*^2^ < 0.01.

**TABLE 1:** Hit rate (%) and reaction time (ms) to targets in the color-matching task.

	Hit rate (%)		RT (ms)	
Orthographic knowledge	Real	Pseudo	Real	Pseudo
High	94 (2.12)	95 (1.32)	506 (20.31)	499 (18.44)
Low	92 (2.39)	93 (1.90)	555 (20.73)	557 (19.58)

*Numbers in the parentheses are standard errors of the mean.*

### Event-Related Potential Data

**FIGURE 4:**
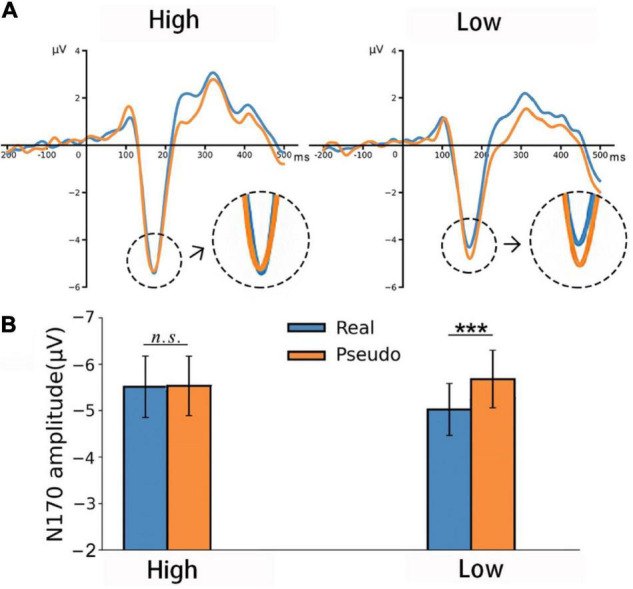
**(A)** The ERP waveforms in response to real and pseudo characters in the P7 channel. **(B)** Bar plots comparing the N170 amplitudes for pseudo and real characters in the high and low orthographic knowledge groups in P7 channel. ***p < 0.001.

The EEG data were recorded for the one-back color-matching task to reveal possible automatic predictions in visual word processing. In the analysis of the EEG data, we focus on the early ERP components, notably P1 and N170. Figure 4A illustrates the ERP waveforms in P7 channel.

#### The P1 Component

Analysis of the P1 amplitudes, with variables Stimulus (real vs. pseudo character), Hemisphere (left vs. right), and Group (high vs. low orthographic knowledge), revealed a significant main effect of Hemisphere, *F*(1,45) = 14.28, *p* < 0.001, η*_*p*_*^2^ = 0.21; the P1 amplitude was larger in the right (P8) than in the left (P7) hemisphere (MD = 1.30). No other main effect or interaction was significant, all *F*’s < 1.76, all *p*’s > 0.14, all η*_*p*_*^2^ < 0.07.

The P1 peak latencies are reported in Table 2. Analysis of the P1 latency also revealed a main effect for Hemisphere, *F*(1,45) = 6.82, *p* < 0.05, η*_*p*_*^2^ = 0.21; the P1 latency was shorter in the left than in the right hemisphere, MD = 4.00, *p* < 0.05. No other main effect or interaction was significant, all *F*’s < 1.71, all *p*’s > 0.18, all η*_*p*_*^2^ < 0.23.

**TABLE 2:** The P1 and N170 latency (ms) for real and pseudo characters.

	P1 latency (ms)		N170 latency (ms)	
Orthographic knowledge	Real	Pseudo	Real	Pseudo
High	81 (4.85)	94 (3.93)	173 (4.35)	177 (3.69)
Low	88 (4.34)	101 (3.36)	174 (2.21)	173 (2.43)

*Standard errors of the means are given in the parentheses.*

#### The N170 Component

Analysis of the N170 amplitudes revealed a main effect for Hemisphere, *F*(1,45) = 5.39, *p* = 0.02, η*_*p*_*^2^ = 0.12, and a significant three-way interaction, *F*(1,45) = 4.74, *p* = 0.01, η*_*p*_*^2^ = 0.13. No other main effect or interaction was significant, all *F*’s < 2.6, all *p*’s > 0.15, all η*_*p*_*^2^ < 0.27. Further analysis revealed that, in the high orthographic knowledge group, there was no reliable difference between real and pseudo characters in both hemispheres, all MD < 0.25, all *p* > 0.24. In the low orthographic knowledge group, the N170 amplitude was significantly higher for pseudo than for real characters in the left hemisphere, MD = 0.59, *p* < 0.001 (see Figure 4B); however, no difference was detected in the right hemisphere, MD = 0.15, *p* = 0.89.

The N170 peak latencies are reported in Table 2. The analysis revealed a significant main effect for Stimulus, *F*(1,45) = 5.65, *p* = 0.02, η*_*p*_*^2^ = 0.17; the N170 peak latency was longer for pseudo than for real characters, MD = 2.36, *p* = 0.02. No other main effect or interaction was significant, all *F*’s < 3.70, all *p*’s > 0.13, all η*_*p*_*^2^ < 0.02.

## Discussion

The present study was set out to examine automatic (non-strategic) predictions and the underlying neural dynamics in visual word processing. The participants were grouped based on how likely they misclassified pseudo characters as real ones. EEGs were recorded when the participants viewed pseudo and real characters in a one-back color matching task. The N170 response to pseudo and real characters was largely the same in participants who were less likely to misclassify pseudo characters as real characters. However, the N170 response to pseudo characters was stronger in participants who were more likely to misclassify pseudo characters as real ones. These results show that word-like visual stimuli would lead to prediction errors that modulate the early stages of visual word processing.

In line with previous findings (Davis et al., 2009; Yang et al., 2012; Taha and Azaizah-Seh, 2017), the present lexical decision task showed that the ability in identifying pseudo characters varied greatly among individuals. According to the “interactive account” (Price and Devlin, 2011), predictions are generated in high-level language areas and automatically feed to vOT. Prediction errors would occur if the visual input and top-down predictions do not match. In the present study, pseudo characters would evoke no or few prediction errors in participants who are less likely to classify pseudo characters as real ones. However, pseudo characters would evoke more prediction errors in participants who are likely to misclassify pseudo characters as real ones. Participants in the high orthographic knowledge group showed no difference in N170 response to real and pseudo characters. The reason being that pseudo characters would not trigger top-down expectations, leading to no or little prediction error; for real characters, the bottom-up visual inputs well match the top-down predictions and thus there was few prediction errors as well (Price and Devlin, 2011; Zhao et al., 2019). Consequently, no N170 difference was found between real and pseudo characters. For the low orthographic knowledge group, however, word-like stimuli (real and pseudo characters) would both trigger top-down predictions (Yum et al., 2014; Zhao et al., 2019). The top-down predictions evoked by real words match the bottom-up visual inputs, and there would be no or few prediction errors (Price and Devlin, 2011; Zhao et al., 2019). Therefore, the top-down predictions evoked by pseudo character do not match bottom-up visual inputs and would lead to prediction errors. As a result, stronger N170 responses were observed for pseudo characters.

The top-down predictions involved in visual word processing can be strategic and vary with task demand (Price and Devlin, 2011). It has been demonstrated in a vast number of studies that the neural activation evoked by visual word forms varies with task demands (Van Berkum et al., 2005; Dambacher et al., 2009; Dikker et al., 2010; Brothers et al., 2015; Wang and Maurer, 2017; for a review, see Nieuwland, 2019). For instance, word-like stimuli evoke stronger neural response in phonological than in orthographic lexical decision task (Twomey et al., 2011). Wang and Maurer (2020) examined the N170 response to visual stimuli in a category expectation task. The results showed that Korean characters evoked a stronger N170 response in native Chinese speakers when they expected a Chinese character but saw an unlearned Korean character. However, no N170 difference was found between Chinese characters and Korean characters, when the participants expected a Korean character but saw a Chinese character. An elevated N170 response is frequently accompanied by improvements in behavioral performance, e.g., faster identification of target letters embedded in native than in non-native words (Maurer et al., 2008) or faster response to strings of alphabetic letters than to strings of symbols (Maurer et al., 2005; Proverbio et al., 2008). So, in these studies, the elevated N170 responses to orthographic stimuli is partly contributed to by task-related or strategic factors (Strijkers et al., 2010; Dehaene and Cohen, 2011; Wang and Maurer, 2017). In the color matching task tested in the present study, however, there was no difference in behavioral performance for real and pseudo characters. It is quite unlikely that results of the present study were confounded by task-related or strategic factors. The stronger N170 response to pseudo characters in the low orthographic knowledge group was most likely due to automatic predictions.

Zhao et al. (2019) showed that, as word knowledge increases in children, more top-down information is automatically activated to aid efficient processing of the orthographic characteristics of visual inputs. The most important contribution of the present work is that we demonstrated automatic predictions in visual word processing in skilled adult readers, providing unambiguous support to the “interactive account.” However, as no language information was needed to complete the color matching task in the present study, the extent to which strategic and automatic (non-strategic) predictions are involved in language-relevant tasks remains unclear. Neural responses related to orthographic feature processing is seen in the left fusiform gyrus (LFG), which overlaps vOT. The EEG technique used in the present study does not have the spatial precision needed to trace the N170 effect to vOT. A high-resolution imaging technique like fMRI is needed to further locate the neural substrates underlying automatic predictions in visual word processing.

## Data Availability Statement

The data used to support the findings of this study are available from the corresponding author upon request.

## Ethics Statement

The studies involving human participants were reviewed and approved by local ethics committee at Hangzhou Normal University. The patients/participants provided their written informed consent to participate in this study.

## Author Contributions

JZ, HY, ZH, SY, and LX designed the experiments. ZH and SY collected the data. LX provided the data analysis tools. ZH, SY, LX, and JZ analyzed the data. ZH and SY drafted the manuscript. JZ, LX, HY, and YL provided the critical revisions. All authors read and approved the submitted version.

## Conflict of Interest

The authors declare that the research was conducted in the absence of any commercial or financial relationships that could be construed as a potential conflict of interest.

## Publisher’s Note

All claims expressed in this article are solely those of the authors and do not necessarily represent those of their affiliated organizations, or those of the publisher, the editors and the reviewers. Any product that may be evaluated in this article, or claim that may be made by its manufacturer, is not guaranteed or endorsed by the publisher.

## References

[B1] Apel K. (2011). What is orthographic knowledge?. *Lang. Speech Hear. Serv. Sch.* 42 592–603. 10.1044/0161-1461(2011/10-0085) 21844399

[B2] Bentin S. Mouchetant-Rostaing Y. Giard M. H. Echallier J. F. Pernier J. (1999). ERP manifestations of processing printed words at different psycholinguistic levels: time course and scalp distribution. *J. Cogn. Neurosci.* 11 235–260. 10.1162/089892999563373 10402254

[B3] Brem S. Halder P. Bucher K. Summers P. Martin E. Brandeis D. (2009). Tuning of the visual word processing system: distinct developmental ERP and fMRI effects. *Hum. Brain Mapp.* 30 1833–1844. 10.1002/hbm.20751 19288464 PMC6871060

[B4] Brothers T. Swaab T. Y. Traxler M. J. (2015). Effects of prediction and contextual support on lexical processing: prediction takes precedence. *Cognition* 136 135–149. 10.1016/j.cognition.2014.10.017 25497522 PMC4308503

[B5] Cao X. H. Zhang H. T. (2011). Change in subtle N170 specialization in response to Chinese characters and pseudocharacters. *Percept. Mot. Skills* 113 365–376. 10.2466/04.22.24.28.PMS.113.5.365-376 22185051

[B6] Chaumon M. Bishop D. V. M. Busch N. A. (2015). A practical guide to the selection of independent components of the electroencephalogram for artifact correction. *J. Neurosci. Methods* 250 47–63. 10.1016/j.jneumeth.2015.02.025 25791012

[B7] Dambacher M. Rolfs M. Göllner K. Kliegl R. Jacobs A. M. (2009). Event-related potentials reveal rapid verification of predicted visual input. *PLoS One* 4:e5047. 10.1371/journal.pone.0005047 19333386 PMC2659434

[B8] Davis C. J. Perea M. Acha J. (2009). Re(de)fining the orthographic neighborhood: the role of addition and deletion neighbors in lexical decision and reading. *J. Exp. Psychol.* 35 1550–1570. 10.1037/a0014253 19803656

[B9] Dehaene S. Cohen L. (2011). The unique role of the visual word form area in reading. *Trends Cogn. Sci*. 15, 254–262. 10.1016/j.tics.2011.04.003 21592844

[B10] Delorme A. Makeig S. (2004). EEGLAB: an open source toolbox for analysis of single-trial EEG dynamics including independent component analysis. *J. Neurosci. Methods* 134 9–21. 10.1016/j.jneumeth.2003.10.009 15102499

[B11] Delorme A. Palmer J. Onton J. Oostenveld R. Makeig S. (2012). Independent EEG sources are dipolar. *PLoS One* 7:e30135. 10.1371/journal.pone.0030135 22355308 PMC3280242

[B12] Devlin J. T. Jamison H. L. Gonnerman L. M. Matthews P. M. (2006). The role of the posterior fusiform gyrus in reading. *J. Cogn. Neurosci.* 18 911–922. 10.1162/jocn.2006.18.6.911 16839299 PMC1524880

[B13] Dikker S. Rabagliati H. Farmer T. A. Pylkkänen L. (2010). Early occipital sensitivity to syntactic category is based on form typicality. *Psychol. Sci.* 21 629–634. 10.1177/0956797610367751 20483838

[B14] Eberhard-Moscicka A. K. Jost L. B. Raith M. Maurer U. (2015). Neurocognitive mechanisms of learning to read: print tuning in beginning readers related to word-reading fluency and semantics but not phonology. *Dev. Sci.* 18 106–118. 10.1111/desc.12189 24863157

[B15] Friston K. (2010). The free-energy principle: a unified brain theory?. *Nat. Rev. Neurosci.* 11 127–138. 10.1038/nrn2787 20068583

[B16] Hinton G. E. (2007). Learning multiple layers of representation. *Trends Cogn. Sci.* 11 428–434. 10.1016/j.tics.2007.09.004 17921042

[B17] Lin S. E. Chen H. C. Zhao J. Li S. He S. Weng X. C. (2011). Left-lateralized N170 response to unpronounceable pseudo but not false Chinese characters-the key role of orthography. *Neuroscience* 190 200–206. 10.1016/j.neuroscience.2011.05.071 21704128

[B18] Liu Y. Wang M. Perfetti C. A. (2007). Threshold-style processing of chinese characters for adult second-language learners. *Mem. Cogn.* 35 471–480. 10.3758/BF03193287 17691146

[B19] Loh E. K. Y. Liao X. Leung S. O. (2018). Acquisition of orthographic knowledge: developmental difference among learners with Chinese as a second language (CSL). *System* 74 206–216. 10.1016/j.system.2018.03.018

[B20] Lupker S. J. Pexman P. M. (2010). Making things difficult in lexical decision: the impact of pseudohomophones and transposed-letter nonwords on frequency and semantic priming effects. *J. Exp. Psychol. Learn. Mem. Cogn.* 36 1267–1289. 10.1037/a0020125 20804296

[B21] Maurer U. Brandeis D. McCandliss B. D. (2005). Fast, visual specialization for reading in English revealed by the topography of the N170 ERP response. *Behav. Brain Funct.* 1:13. 10.1186/1744-9081-1-13 16091138 PMC1208852

[B22] Maurer U. Brem S. Kranz F. Bucher K. Benz R. Halder P. (2006). Coarse neural tuning for print peaks when children learn to read. *NeuroImage* 33 749–758. 10.1016/j.neuroimage.2006.06.025 16920367

[B23] Maurer U. Rossion B. McCandliss B. D. (2008). Category specificity in early perception: face and word N170 responses differ in both lateralization and habituation properties. *Front. Hum. Neurosci.* 2:18. 10.3389/neuro.09.018.2008 19129939 PMC2614860

[B24] McCandliss B. D. Cohen L. Dehaene S. (2003). The visual word form area: expertise for reading in the fusiform gyrus. *Trends Cogn. Sci.* 7 293–299. 10.1016/S1364-6613(03)00134-7 12860187

[B25] Nieuwland M. S. (2019). Do ‘early’ brain responses reveal word form prediction during language comprehension? A critical review. *Neurosci. Biobehav. Rev.* 96 367–400. 10.1016/j.neubiorev.2018.11.019 30621862

[B26] Price C. J. Devlin J. T. (2011). The Interactive Account of ventral occipitotemporal contributions to reading. *Trends Cogn. Sci.* 15 246–253. 10.1016/j.tics.2011.04.001 21549634 PMC3223525

[B27] Proverbio A. M. Zani A. Adorni R. (2008). The left fusiform area is affected by written frequency of words. *Neuropsychologia* 46 2292–2299. 10.1016/j.neuropsychologia.2008.03.024 18485421

[B28] Reinke K. Fernandes M. Schwindt G. O’Craven K. Grady C. L. (2008). Functional specificity of the visual word form area: general activation for words and symbols but specific network activation for words. *Brain Lang.* 104 180–189. 10.1016/j.bandl.2007.04.006 17531309

[B29] Rossion B. Joyce C. A. Cottrell G. W. Tarr M. J. (2003). Early lateralization and orientation tuning for face, word, and object processing in the visual cortex. *NeuroImage* 20 1609–1624. 10.1016/j.neuroimage.2003.07.010 14642472

[B30] Shtyrov Y. Goryainova G. Tugin S. Ossadtchi A. Shestakova A. (2013). Automatic processing of unattended lexical information in visual oddball presentation: neurophysiological evidence. *Front. Hum. Neurosci.* 7:421. 10.3389/fnhum.2013.00421 23950740 PMC3738864

[B31] Strijkers K. Bertrand D. Grainger J. (2010). Seeing the same words differently: the time course of automaticity and top – down intention in reading. *J. Cogn. Neurosci.* 27 1542–1551. 10.1162/jocn_a_00797 25761003

[B32] Taha H. Azaizah-Seh H. (2017). Visual word recognition and vowelization in Arabic: new evidence from lexical decision task performances. *Cogn. Process.* 18 521–527. 10.1007/s10339-017-0830-9 28840361

[B33] Tong X. Mcbride-Chang C. Shu H. Wong A. M. Y. (2009). Morphological awareness, orthographic knowledge, and spelling errors: keys to understanding early chinese literacy acquisition. *Sci. Stud. Read.* 13 426–452. 10.1080/10888430903162910

[B34] Twomey T. Kawabata Duncan K. J. Price C. J. Devlin J. T. (2011). Top-down modulation of ventral occipito-temporal responses during visual word recognition. *NeuroImage* 55 1242–1251. 10.1016/j.neuroimage.2011.01.001 21232615 PMC3221051

[B35] Van Berkum J. J. A. Brown C. M. Zwitserlood P. Kooijman V. Hagoort P. (2005). Anticipating upcoming words in discourse: evidence from ERPs and reading times. *J. Exp. Psychol. Learn. Mem. Cogn.* 31 443–467. 10.1037/0278-7393.31.3.443 15910130

[B36] Wang F. Maurer U. (2017). Top-down modulation of early print-tuned neural activity in reading. *Neuropsychologia* 102 29–38. 10.1016/j.neuropsychologia.2017.05.028 28576569

[B37] Wang F. Maurer U. (2020). Interaction of top-down category-level expectation and bottom-up sensory input in early stages of visual-orthographic processing. *Neuropsychologia* 137 107299. 10.1016/j.neuropsychologia.2019.107299 31821829

[B38] Woodhead Z. V. J. Brownsett S. L. E. Dhanjal N. S. Beckmann C. Wise R. J. S. (2011). The visual word form system in context. *J. Neurosci.* 31 193–199. 10.1523/JNEUROSCI.2705-10.2011 21209204 PMC6622763

[B39] Xue L. Maurer U. Weng X. Zhao J. (2019). Familiarity with visual forms contributes to a left-lateralized and increased N170 response for Chinese characters. *Neuropsychologia* 134:107194. 10.1016/j.neuropsychologia.2019.107194 31542360

[B40] Yang J. Wang X. Shu H. Zevin J. D. (2012). Task by stimulus interactions in brain responses during Chinese character processing. *NeuroImage* 60 979–990. 10.1016/j.neuroimage.2012.01.036 22248577 PMC3508434

[B41] Yum Y. N. Law S. P. Su I. F. Lau K. Y. D. Mo K. N. (2014). An ERP study of effects of regularity and consistency in delayed naming and lexicality judgment in a logographic writing system. *Front. Psychol.* 5:315. 10.3389/fpsyg.2014.00315 24782812 PMC3995054

[B42] Zhao J. Li S. Lin S. E. Cao X. H. He S. Weng X. C. (2012). Selectivity of N170 in the left hemisphere as an electrophysiological marker for expertise in reading Chinese. *Neurosci. Bull.* 28 577–584. 10.1007/s12264-012-1274-y 23054635 PMC5561926

[B43] Zhao J. Maurer U. He S. Weng X. (2019). Development of neural specialization for print: evidence for predictive coding in visual word recognition. *PLoS Biol.* 17:e3000474. 10.1371/journal.pbio.3000474 31600192 PMC6805000

